# Fecal Microbiota Transplantation Reduces Pathology and Improves Cognition in a Mouse Model of Alzheimer’s Disease

**DOI:** 10.3390/cells12010119

**Published:** 2022-12-28

**Authors:** Shalini Elangovan, Thomas J. Borody, R. M. Damian Holsinger

**Affiliations:** 1 Neuroscience, School of Medical Sciences, Faculty of Medicine and Health, The University of Sydney, Camperdown, NSW 2050, Australia; 2 Centre for Digestive Diseases, Level 1, 229 Great North Road, Five Dock, NSW 2046, Australia; 3 Laboratory of Molecular Neuroscience and Dementia, School of Medical Sciences, Faculty of Medicine and Health, The University of Sydney, Camperdown, NSW 2006, Australia

**Keywords:** fecal microbiota transplants, Alzheimer’s disease, gut microbiome, 5xFAD

## Abstract

Characterized by the presence of amyloid plaques, neurofibrillary tangles and neuroinflammation, Alzheimer’s disease (AD) is a progressive neurodegenerative disorder with no known treatment or cure. Global disease projections warrant an urgent and rapid therapeutic for the treatment of this devastating disease. Fecal microbiota transplantation (FMT) is a widely accepted and safely used treatment for recurrent *Clostridium difficile* infection and other metabolic diseases such as diabetes mellitus. FMT has also been demonstrated to be a possible AD therapeutic. We examined the potential of FMT for the treatment of AD in a robust, mouse model of the disease and report that a brief, 7-day treatment regimen demonstrated ‘plaque-busting’ and behavior-modifying effects in treated 5xFAD mice. Importantly, we show that donor age plays an important role in the efficacy of the treatment and these findings warrant further investigation in human trials.

## 1. Introduction

Alzheimer’s disease (AD) is a progressive neurodegenerative condition resulting in cognitive, behavioral and functional decline with a pre-dementia phase termed mild cognitive impairment (MCI) [1,2]. The pathological hallmarks of AD include extracellular deposition of amyloid-β (Aβ) peptide, intracellular accumulation of the microtubule-associated protein tau into neurofibrillary tangles (NFT), with both possibly involved in a feedback loop where each protein causes further deposition of the other [3,4], synaptic degeneration [5,6] and neuroinflammation [7]. Although genetic risk factors have been well-established in EOAD [6,8,9,10], various other causal mechanisms have been investigated and proven to contribute to the development of AD, including obesity [11], increased plasma cholesterol and type 2 diabetes mellitus [12,13].

The human microbiome has been described to have a distinct, complex role in the pathophysiology of multiple diseases [14]. Inter-individual variation [15] as well as age-dependent changes [16] in the human gut microbiome have been described, and gut microbial involvement has been suggested in neurodevelopmental [17,18], autoimmune diseases [19], movement disorders [20,21] and neurodegeneration [21,22,23]. Intestinal microbial composition of AD patients differs from healthy individuals, with AD patients observed to have increased Bacteroidetes and decreased Firmicutes and *Bifidobacterium* levels [24]. Modulating intestinal microbial composition has been shown to decrease Alzheimer’s symptoms in mice [7,25,26]. Although the involvement of intestinal microbiota in the development and progression of AD has been established, its exact role has not been delineated despite early evidence of inflammatory pathway involvement [7]. Considering the changes that have been reported in the gut microbiome of AD patients, replacing or replenishing the phyla could have therapeutic implications.

FMT involves the transfer of fecal material from a healthy donor to a recipient with the aim of normalizing composition and therefore function of intestinal microbial populations [27]. FMT has been successfully used to treat recurrent *Clostridium difficile* infections [28] with expansions of its application into the treatment of other gastrointestinal disorders [29]. The efficacy of FMT has also been described in reducing intestinal dysbiosis in Parkinson’s mice [10] and alleviating multiple neurological disorders [30,31]. FMT has also been demonstrated to reduce Alzheimer pathology in a transgenic mouse model following pre-FMT antibiotic treatment to void the gut of its host microbiome [25]. Crucially, immense potential for FMT as an AD therapeutic has been demonstrated [32,33,34,35,36]. We show a donor age-dependent relationship between FMT and reduction of Alzheimer’s pathology as well as the efficacy of FMT in the absence of antibiotic administration.

## 2. Materials and Methods

### 2.1. Animals and Treatment

To explore the impact of FMT in a murine model of AD, we performed FMT via oral gavage in 16 Old (30–32-week-old) 5xFAD recipient mice using fecal matter from healthy B6SJL wildtype donor mice (either 10–12- or 30–32-week-old) over a period of seven days. We also assessed the impact of FMT in 16 Young (10–12-week-old) 5xFAD mice. Mice ages were determined based on the fact that deposition of amyloid plaques commence at 6–8 weeks of age in the 5xFAD mice. Observations in our 5xFAD colony revealed that there is substantive deposition of plaques at 10–12 weeks. The 5xFAD mouse brain is laden with plaques at the age of 30–32 weeks and mice of this age represent the late stages of the disease. Duration of incubation was informed by previously described FMT procedures in published literature [37,38,39,40].

Ethics approval was obtained from the University of Sydney Animal Ethics Committee (AEC Number: 2017/1285) and all procedures were in accordance with institutional guidelines. Heterozygous male and female 5xFAD mice overexpressing three human APP (Swedish, Florida and London) Familial AD (FAD) mutations as well as two human Presenilin 1 mutations were bred under standard laboratory conditions at the Bosch Laboratory Animal Services Facility and were maintained on a 12:12 h light/dark cycle with food and water provided ad libitum. Wild type B6SJL mice served as fecal donors.

**Figure 1:**
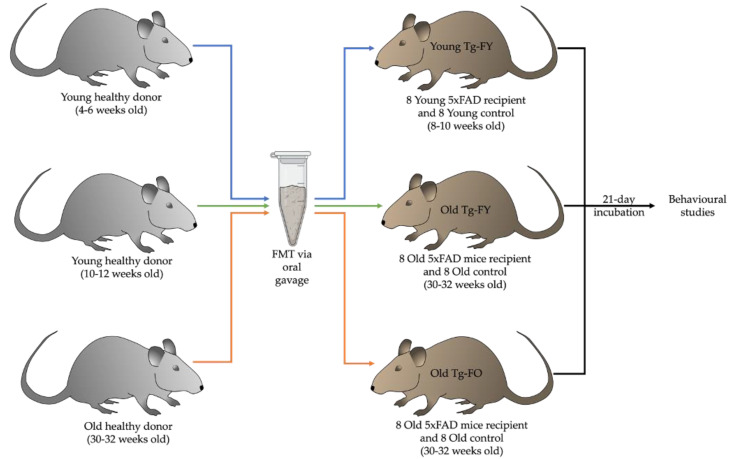
Diagram showing treatment and numbers of healthy (WT) and transgenic mice undergoing FMT via oral gavage. Young Tg-FY: Young Transgenic-Fed Young, Old Tg-FY: Old Transgenic-Fed Young, Old Tg-FO: Old Transgenic-Fed Old.

Half of the old recipient mice received oral gavage of fecal slurry obtained from healthy wild type (WT) donors of the same age (Old Tg-fed old; Old Tg-FO), depicted as orange arrows in Figure 1, and the other half received oral gavage of fecal slurry from healthy, younger donors (Old Tg-fed young; Old Tg-FY), depicted with green arrows in Figure 1. Young recipient mice (8–10 weeks old) received fecal slurry from healthy, younger (4–6-week) donors (Young Tg-fed young; Young Tg-FY), shown using blue arrows in Figure 1. The control 5xFAD recipient mice (8–10 and 30–32 weeks old) were gavaged with normal saline (Old Tg-Control and Young Tg-Control). Following an incubation period of 21 days, mice were subjected to a battery of behavioral tests to investigate the effects of FMT on cognition, after which cerebral Aβ levels were evaluated. Behavioral studies were also performed on healthy wildtype mice with no AD phenotype which served as controls for comparison of normal age-matched behavior (Old-WT and Young-WT) and provided baseline approximate values for behavioral tests.

Fresh fecal matter was collected daily from healthy wildtype donors aged 4–6 weeks (for Young Tg-FY), 10–12 (Old Tg-FY) and 30–32 (Old Tg-FO) weeks. Fecal matter was homogenized using a micro-pestle in 0.9% sterile saline solution. The slurry was then centrifuged at 1000× *g* to collect the supernatant. This procedure was repeated once, and the supernatants pooled to provide a final 50 mg/mL fecal slurry. The pellet was discarded whilst the supernatant was retained and used for oral gavage. Each treated mouse received 200 μL of supernatant via oral gavage using a 22 G oral gavage needle daily for 7 days with control animals receiving 200 μL of sterile saline. Mice were monitored for general health and weight during the gavage procedure. Following oral gavage, all mice were subjected to a 21-day incubation period with chow and water available ad libitum.

### 2.2. Behavioral Studies

#### 2.2.1. Elevated Plus Maze

Emotional symptoms such as anxiety arise from Aβ and tau accumulation in the amygdala [40] and can be assessed by means of the elevated plus maze (EPM) [41]. During the EPM, mice were placed in the middle of the platform and allowed to explore the maze for 5 min. An arm entry was recorded when 80% of the mouse’s body was in the arm. Stretch-attend posture was not included as an arm entry. Time spent in the central platform was not recorded. Arm entries were measured and analyzed as a ratio of entries into closed arms to entries into open arms. The duration of time spent in the open and closed arm each was measured in seconds and analyzed as a percentage of time spent in closed arms. The percentage of time spent in the closed arm and ratio of closed arm entry to open arm entry was measured as indicators of anxiety.

#### 2.2.2. Novel Object Recognition

Dorsal hippocampal involvement in recognition and spatial memory formation and hippocampal degeneration during the course of AD are evaluated by employing the novel object recognition (NOR) paradigm [42]. The NOR involves exposure to an object (Familiar Object) for 5 min in Phase 1, followed by an interval spent in the home cage and subsequently, exposure to the Familiar Object and a Novel Object for 5 min. The time spent interacting with the Novel Object and Familiar Object in Phase 2 was measured separately in seconds. To understand object preference in the mice, we calculated a Discrimination Index (DI) which assigns a numerical value indicating object preference. The more negative the number, the less preference there is for the Novel Object.

#### 2.2.3. Forced Alternation Y-Maze

Synaptic degeneration of field CA1 in the hippocampus, which receives innervation from pyramidal neurons from the entorhinal cortex, results in spatial memory decline in AD mice [43,44], which is also apparent in hippocampal atrophy and neuron loss seen in AD patients [45] and is evaluated in mice in the form of a forced alternation Y-maze (FAY) [41]. The procedure for the FAY is similar to the NOR, involving an initial phase where mice were given 5 min to explore the Y-maze with a single arm blocked from entry. In Phase 2, mice have access to all 3 arms. The duration of time spent in the maze was analyzed for Phase 2 and the time spent in each arm in seconds as well the number of arm entries were recorded. An arm entry was counted when 80% of the mouse’s body entered the arm. Stretch-attend posture was not included as an arm entry. Time spent in the central platform was not counted. The FAY results were analyzed in the form of time spent in the Novel Arm and ratio of Novel to Other arm entries.

### 2.3. Amyloid Quantification

Aβ plaque loading was evaluated using Thioflavin S staining and analyzed using unbiased stereological methods as the percentage area of cortical amyloid plaque and quantified as the number of cortical plaques using open-source software CellProfiler [46]. Thioflavin S binds to amyloid and is a widely used method for cortical Aβ staining [47].

On CellProfiler, a customized plate template was used to mask slide scanner images of brain slices and a pipeline was used to distinguish and draw dividing lines between clumped objects using intensity. Whole brain slices were analyzed using the mask. Primary objects identified on masked images were cortical plaques. Cortical Aβ plaque loading was retrieved from the ‘Area covered by objects’ parameter and plaques were stratified and binned according to size in μm^2^.

### 2.4. Cognition Score

To reflect the correlation of Aβ plaque load and cognitive performance, a compounded Cognition Score (CS) of the behavioral study results was calculated for each subject of each group of the behavioral study results and plotted against the mean percentage area of amyloid plaque, or amyloid plaque loading (AP).

### 2.5. Statistical Analysis

Statistical analysis was carried out using the SPSS Statistics Package (version 24; IBM). The Shapiro–Wilk test of normality, two-way Student’s *t*-tests to compare means of experimental results between Old and Young control and treated groups and analysis of variance (ANOVA) to compare means of multiple groups were used to detect statistical differences between groups. Results are reported as mean ± SEM and corresponding *p*-value to indicate statistical significance.

## 3. Results

Following treatment, Old Tg-FY mice were found to display improved overall cognition whilst Old Tg-FO also displayed some cognitive improvement. The increasing trend in cognitive improvement observed in the Old groups correlate with decreasing Aβ load. Young Tg-FY mice failed to demonstrate significant behavioral changes despite a reduction in Aβ loading.

### 3.1. Behavioural Studies

#### 3.1.1. Elevated Plus Maze

**Figure 2:**
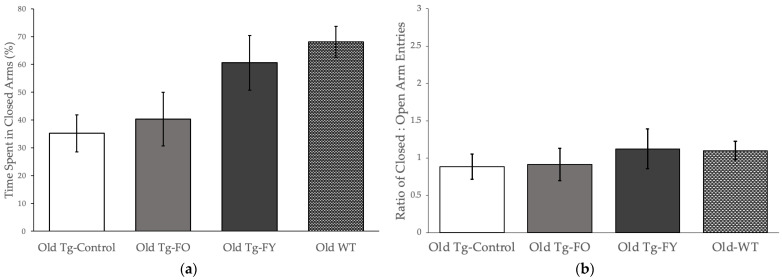

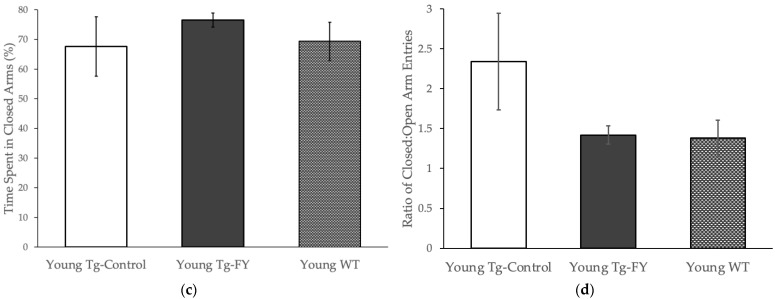
FMT treated mice show normalization of behavior approaching control levels on the elevated plus maze. Old Tg-FY groups (*n* = 8) show improvement in anxiety-related behavior with increased (**a**) Percentage of time spent in closed arms and (**b**) Ratio of Closed:Open arm entries. Young Tg-FY group (*n* = 8) showed overall decreased anxiety with (**c**) a small increase in Percentage of time spent in closed arms and (**d**) decreased Ratio of Closed:Open arm entries. Data is presented as mean ± SEM. Young Tg-FY: Young Transgenic-Fed Young, Young Tg-Control: Young Transgenic Control, Old Tg-FY: Old Transgenic-Fed Young, Old Tg-FO: Old Transgenic-Fed Old, Old Tg-Control: Old Transgenic Control, Young WT: Young Wildtype, Old WT: Old Wildtype.

Mice in the Old Tg-FO group spent an average 60% of their time in the open arms, similar to the Old Tg-Control mice who spent ~65% in the open arms of the maze (*p* = 0.687; Students *t*-test), displaying low levels of anxiety (Figure 2a, Old Tg-Controls vs. Old Tg-FO). Comparatively, Old Tg-FY mice displayed higher levels of anxiety (*p* = 0.056) compared to Old Tg-Control (Figure 2a, Old Tg-FY vs. Old Tg-Controls), spending only ~39% of their time in the open arms of the EPM, similar to WT mice, used for comparison to age-matched behavior, who spent only 32% of their time in the open arms. Comparing the number of open to close arm entries (Figure 2b), where an increase in the number of open arm entries depicts anti-anxiety behavior, we found no significant differences (*p* = 0.192, ANOVA) in the number of entries between the three old Tg groups indicating that the Old Tg-FY mice spent longer periods of time in the closed arm of the maze and less time exploring their environment, typical of anxiety-related behavior.

Young Tg-FY mice spent an average 76% of their time in the closed arm of the maze, similar in duration to Young Tg-Control (70%) and Young WT (66%) mice (Figure 2c; One-way ANOVA *p* = 0.192). Young Tg-Control mice made many more entries into the closed arms compared to Young Tg-FY (and Young WT) mice (Figure 2d), probably reflective of hyperactive behavior observed in the transgenic mice and a potential normalization following FMT from young WT (Figure 2d, Young Tg-FY vs. Young WT) mice that should be further explored.

#### 3.1.2. Novel Object Recognition

**Figure 3:**
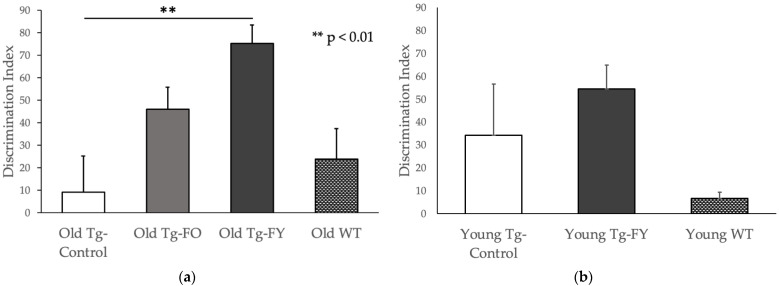
FMT treated mice show improved recognition and spatial memory levels in Novel Object Recognition test measured using Discrimination Index (DI). (**a**) Old Tg-FO (*n* = 8) and Old Tg-FY (*n* = 8) had increased Discrimination Index (Old Tg-FY *p* < 0.01 compared to Old Tg-Control (*n* = 7)). (**b**) Young Tg-FY also showed improved DI compared to Young Tg-Control (*n* = 8). WT values are presented for comparison only. Data is presented as mean ± SEM. Young Tg-FY: Young Transgenic-Fed Young, Young Tg-Control: Young Transgenic Control, Old Tg-FY: Old Transgenic-Fed Young, Old Tg-FO: Old Transgenic-Fed Old, Old Tg-Control: Old Transgenic Control, Young WT: Young Wildtype, Old WT: Old Wildtype.

Old Tg-FO mice demonstrated more than a two-fold increase in the DI (*p* = 0.065), indicating a greater preference for the Novel Object when compared to Old Tg-Control mice (Figure 3a). Old Tg-FY mice demonstrated a significantly greater, approximately five-fold increase in the DI (*p* = 0.002), indicating a much higher preference for the Novel Object compared to control (Figure 3a). Young Tg-FY mice demonstrated an increased DI compared to the Young Tg-Controls, in contrast to Young-WT (used only to demonstrate ‘normal’ levels) who demonstrated a much lower DI than both Young Tg-FY and Young-Tg Control (Figure 3b). The decreased DI of Young-WT, which was close to zero indicating no preference for either object, could possibly be a product of higher levels of circumstantial neophobia rather than an indication of object preference as WT mice usually show preference to the novel object [47,48].

#### 3.1.3. Forced Alternation Y-Maze

**Figure 4:**
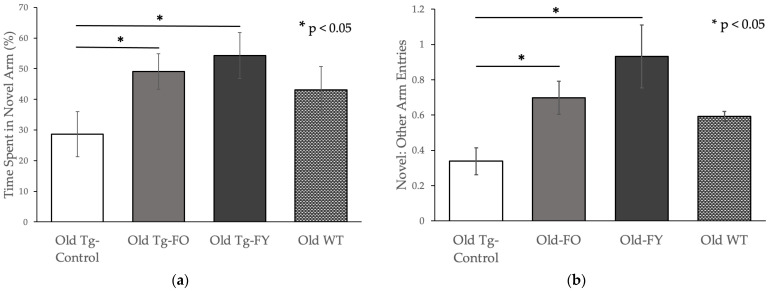

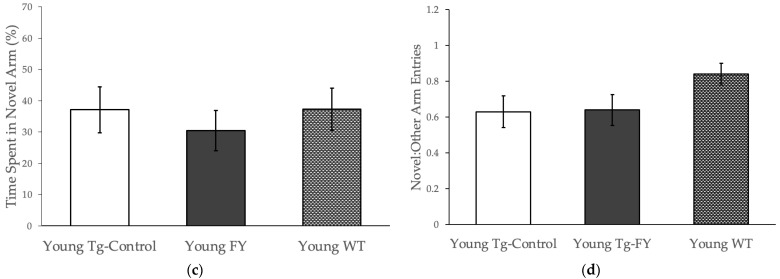
Spatial memory levels improved in Old treated mice in Forced Alternation Y-maze. Data is presented as mean ± SEM. Old Tg-FO (*n* = 8) and Old Tg-FY (*n* = 8) demonstrated increased (**a**) exploration time in Novel arm and (**b**) Ratio of Novel to Open arm entries (*p* < 0.05 compared to Old Tg-Control (*n* = 7)). (**c**,**d**) Young Tg-FY (*n* = 8) did not show improvement in FAY compared to Young Tg-Control (*n* = 7). WT values are presented for comparison only. Young Tg-FY: Young Transgenic-Fed Young, Young Tg-Control: Young Transgenic Control, Old Tg-FY: Old Transgenic-Fed Young, Old Tg-FO: Old Transgenic-Fed Old, Old Tg-Control: Old Transgenic Control, Young WT: Young Wildtype, Old WT: Old Wildtype.

Old Tg-FO animals showed a significant, near two-fold increase (*p* = 0.046) while Old Tg-FY demonstrated a significant two-fold increase (*p* = 0.030) in time spent in the Novel Arm (Figure 4a). Compared to the Old-WT mice who were used for comparison, the Old treated groups spent more time exploring the Novel Arm. When comparing the ratio of Novel:Other arm entries, Old Tg-FO mice also displayed an approximately two-fold, statistically significant increase (*p* = 0.012) in preference for the Novel arm while Old Tg-FY mice demonstrated a nearly three-fold, statistically significant increase (*p* = 0.012) in preference (Figure 4b).

Compared to Old-WT mice who were used for comparison purposes only, both groups of Old-treated animals showed a higher sense of exploratory behavior, demonstrated by a greater ratio of Novel:Other arm entries (Figure 4b). Old Tg-FO and Old Tg-FY showed remarkable improvement in spatial memory following FMT, with increases in both the percentage of time spent in the Novel Arm and the ratio of Novel arm entries to Other arm entries (Figure 4a,b). When comparing the Young mice, it was noted that the Tg-Control mice spent almost identical amounts of time in the Novel Arm compared to Young WT mice and treatment did not significantly alter this exploratory behavior (*p* = 0.505) (Figure 4c), reflected by similar ratios of Novel:Other arm entries (*p* = 0.944; Figure 4d).

### 3.2. Amyloid Quantification

**Figure 5:**
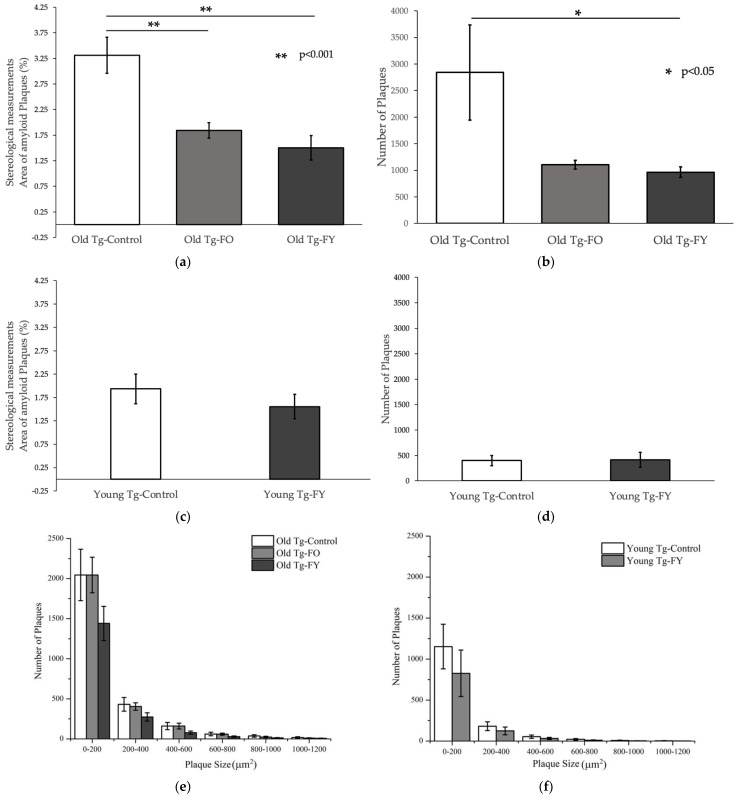

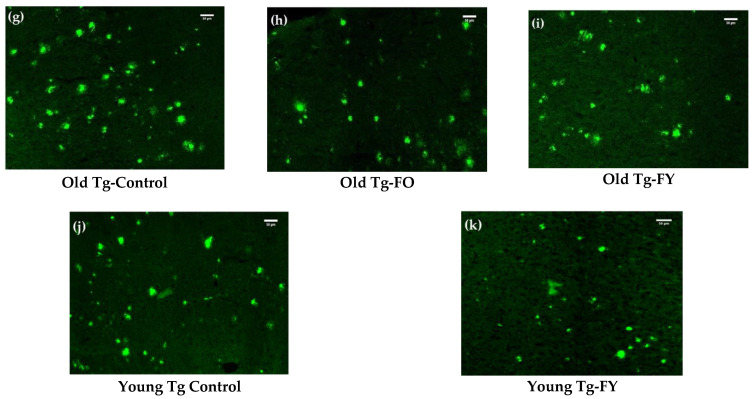
FMT resulted in decreased amyloid plaque burden in treated mice as seen in Thioflavin S—stained brain sections. (**a**) Old Tg-FO and Old Tg-FY both had reduced percentage amyloid plaque with a nearly 2-fold decrease in Old Tg-FO (*p* < 0.01) and Old Tg-FY mice (*p* < 0.01) as well as (**b**) fewer plaques in both groups of treated mice (Old Tg-FY *p* < 0.05). (**c**) Young Tg-FY mice also showed a reduced percentage area of amyloid plaques with (**d**) no difference seen in the number of plaques. (**e**) Old Tg-FY mice showed decreased numbers of varying plaque sizes as compared to both Old Tg-Control and Old Tg-FO while (**f**) Young Tg-FY also showed decreased numbers of all plaque sizes compared to Young Tg-Controls. (**h**) Old Tg-FO mice and (**i**) Old Tg-FY had lesser amyloid plaque burden compared to (**g**) Old Tg-Controls. Mature, compact plaques were more diffuse with less defined outlines following FMT. Although (**k**) Young Tg-FY mice did not demonstrate decreased plaque burden compared to (**j**) Young Tg-Control mice, plaque dispersal appeared similar to the Old treated mice. Data is presented as mean ± SEM. Scale bar = 50 μm. Young Tg-FY: Young Transgenic-Fed Young, Young Tg-Control: Young Transgenic Control, Old Tg-FY: Old Transgenic-Fed Young, Old Tg-FO: Old Transgenic-Fed Old, Old Tg-Control: Old Transgenic Control.

Old Tg-FO and Old-Tg-FY showed statistically significant reductions (*p* < 0.01 for both; Figure 5a) in cortical amyloid loading when assessed stereologically [45,49,50,51] and compared to Old Tg-Controls. Both Old Tg-FO and Old Tg-FY demonstrated a decrease in the number of quantifiable plaques within the cortex, with Old Tg-FO having a near 2.5-fold decrease (*p* = 0.060) and Old Tg-FY having more than a 2.5-fold decrease (*p* = 0.045) (Figure 5b). Furthermore, Old Tg-FY had a pronounced reduction in the numbers of smaller cortical plaques ranging in 0–800 µm^2^ in size and modest decreases in larger plaque sizes of 800–1200 µm^2^ (Figure 5e). Stereological analysis of Young Tg-FY (*n* = 7) also showed a decrease in the area of amyloid plaque present in the cortex (*p* = 0.359) but similar plaque numbers to Young Tg-Control (Figure 5f). However, examination of mice cortices revealed a visible decrease in Thioflavin S staining intensity in the treated mice (Figure 5k vs. Figure 5j), indicating a decreased rate of accumulation of cortical amyloid plaques resulting in smaller plaques in Young Tg-FY mice (Figure 5k) compared to Young Tg-Controls (Figure 5j). The number of overall plaques, quantified across all sizes were reduced in Young Tg-FY mice compared to Young Tg-Controls (Figure 5e vs. Figure 5f).

### 3.3. Cognition Score

**Figure 6:**
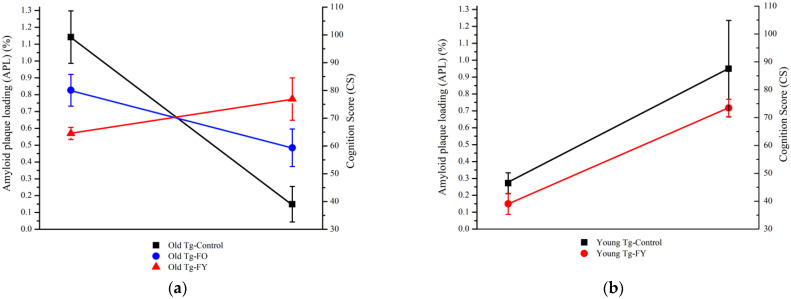
Cognition score based on behavioral study results compared to amyloid plaque burden shows donor age-dependent differences between treated mice groups. (**a**) Mice receiving fecal transplants from younger donors (Old Tg-FY) had higher cognition scores that correlated with lower amyloid loading compared to Old Tg-FO mice that received transplants from age-matched donors. (**b**) Young Tg-FY mice showed marginal decreases in amyloid load following treatment. Young Tg-FY: Young Transgenic-Fed Young, Young Tg-Control: Young Transgenic Control, Old Tg-FY: Old Transgenic-Fed Young, Old Tg-FO: Old Transgenic-Fed Old, Old Tg-Control: Old Transgenic Control.

The decreasing trend of Aβ plaque load across Old Tg-FO and Old-Td-FY is inversely correlated with the trend of improved cognition, which was measured as a compounded Cognition Score of the behavioral study results (CS), indicating the importance of donor age in decreasing amyloid pathology and improving cognition (Figure 6a). The resulting correlation showed that a lower plaque burden in Old Tg-FO and Old Tg-FY resulted in improved cognition compared to Old Tg-Control. Interestingly, Old Tg-FY had a much higher CS and lower amyloid plaque loading (APL) compared to Od Tg-FO, indicating that donor age influences FMT outcomes. Young Tg-FY mice demonstrated marginally decreased APL and CS compared to controls (Figure 6b). The decrease in CS in the Young Tg-FY mice does not mirror the effects seen in older Tg mice and is reflective of the significantly fewer entries made into the open arm of the EMP by young WT mice (*p* = 0.001), an anxiety-associated feature.

## 4. Discussion

Restoring gut microbial composition and reducing dysbiosis is a key function of FMT. We performed a short course of FMT in a robust mouse model of AD and report phenomenal cognitive and pathological changes in old, AD mice.

Young (10–12 week) donor mice have a distinct microbial composition compared to that of Old mice (30–32 week) mice [52]. As such, a microbiome transplantation would be expected to recolonize the recipients gut with the donors’ microbiome. To determine whether such a transplantation would be viable as a treatment for Alzheimer’s disease, we transplanted fecal microbiome from either young or old wildtype mice into old, 5xFAD mice. We also transplanted fecal microbiome from young wildtype mice to young 5xFAD mice as well. The treatment in all groups was performed for 7 days followed by an incubation period of 21 days. At the end of incubation, memory and behavioral tests were performed to evaluate the efficacy of treatment. We employed the Y-maze Forced Alternation test to explore general cognitive function as it is sensitive to hippocampal damage, a Novel Object Recognition task that is used to evaluate cognition, particularly recognition memory, and the elevated plus-maze to study anxiety. These tests revealed significant changes in the treated animals. Old-Tg mice receiving transplants from young wildtype mice showed the most robust and consistent changes with significant alterations observed in the Y-maze (*p* = 0.03), a 5-fold increase in the NOR test (*p* = 0.002), nearly twice as much time in closed arms of the elevated plus maze and 2- to 3-fold decreases in areas occupied by amyloid plaques and plaque numbers in the brain, respectively.

Research has shown that microbiota from aged mice contribute to low-grade inflammation [53], hence, normalizing gut microbe composition to a younger population should result in the reduction of intestinal and systemic inflammation. Consequently, neuroinflammation-associated deterioration, such as the loss of blood-brain-barrier integrity [54], could also be expected to be reversed. Additionally, dysfunctional metabolite levels in AD [55] could also be restored by FMT, culminating in the normalization of these levels and subsequent reduction of pathology. The gut-brain axis, comprising of bidirectional communication between the enteric nervous system (ENS) and the central nervous system (CNS), including neurotransmitter expression, anxiety and stress levels, and cognition is affected by intestinal microbes [56]. We report significantly decreased amyloid burden and improved cognition in the brains of FMT treated mice and conclude that a short course of FMT was able to increase clearance of cortical Aβ, possibly via immune or inflammatory regulation, allowing for improved cognition.

Our study was performed as a ‘proof of principle’ investigation, and we did not envisage such dramatic changes following a short period of treatment. Our results demonstrate that populating a dysbiotic host with ‘normal’ microbiome is capable of significant changes in pathology that in turn, translate to cognitive improvements as well. An additional surprising observation was that these results were obtained without the need for destroying the host microbial environment, a procedure normally performed using antibiotics.

We postulate that the effects on behavior and cognitive correlates observed in our experiments demonstrate that FMT was capable of ‘repairing’ a ‘damaged’ nervous system in a very short period of time. Experiments currently in progress are investigating parameters of treatment that would be most efficacious in translating these findings to the clinic. The hypothalamic-pituitary-adrenal axis is involved in the regulation of stress hormones such as cortisol and is intimately associated with and influenced by the amygdala, which in turn interacts with the hippocampus and hypothalamus. Reduction of dysbiotic intestinal microbiota could restore permeability of the intestinal epithelium and potentially normalize levels of cortisol via the gut-brain axis, resulting in decreased levels of anxiety, especially in Old Tg-FY mice. Furthermore, FMT may allow for increased clearance or renewal of clearance mechanisms of Aβ from the amygdala and hippocampus, amongst other structures, which could result in reduced anxiety. Old Tg-FO and Old Tg-FY groups both had increased DI, indicating improved recognition memory sensitivity [57] for treated groups compared to transgenic controls. Our results indicate that the restoration of hippocampal function (to a limited extent) in treated groups was possibly related to an increased clearance of Aβ from the hippocampus. The hippocampus also expresses and relies heavily on proteins such as brain-derived neurotrophic factor (BDNF), which is known to be decreased in Alzheimer’s disease [58,59]. Here, we showed improved recognition memory in treated animals compared even to age-matched wildtype littermates in the novel object recognition (NOR) task. The forced alternation Y-maze (FAY) results demonstrate that FMT is particularly efficient in improving spatial memory, indicating its potency in restoring hippocampal function. Similar to the EPM and NOR results, the outcome from the FAY task demonstrates that FMT may be boosting Aβ clearance, allowing for improved spatial memory. This topic is contentious amongst the research community, but a recent clinical trial has demonstrated that amyloid plaque-targeting therapies can impede cognitive decline [60].

Young Tg-FY mice showed little change in spatial memory. We attribute this to the age of the treated mice. Young 5xFAD mice commenced treatment at an age of 8–10 weeks, with behavioral tests being performed at an age of 13–15 weeks. Despite the fact that amyloid deposition commences at 7–8 weeks of age in these mice, plaque number and size are substantially lower compared to 30-week-old mice. Additionally, memory correlates are also unaffected at this age compared to age-matched WT littermates. The threshold of Aβ loading and cognitive deficits in the 5xFAD mouse are generally reached at an age of 4–5 months and as such, innate protective clearance mechanisms were most likely still functional in the young mice used in this study. Conversely, as both plaque size and number decreased significantly in old mice that were treated with FMT, overall amyloid burden was decreased, and this translated into improved memory and cognition in the old, treated animals. We postulate that the increased clearance of Aβ could be attributed to FMT-induced renewal of immune-mediated clearance mechanisms or inflammatory regulation [61]. Future studies will need to investigate the glial-mediated response to FMT in these mice and also study potential phagocytic clearance mechanisms that are driven by alterations in the gut microbiome.

Our results also demonstrate a novel finding in relation to FMT, whereby we show that donor age represents an important variable in FMT. Old transgenic mice receiving fecal transplants from 10–12-week-old WT donors, showed significantly improved behavior in the FAY and NOR tests. In addition, amyloid plaque load was significantly reduced in old mice receiving transplants from both age matched as well as young donors. Although performance in the EPM test did not reach significance in Old Tg-FY mice, analysis showed a trend towards normalization (*p* = 0.056). Old 5xFAD mice receiving fecal transplants from age-matched donors also showed somewhat improved recognition and significantly improved spatial memory. Overall, the most robust changes were observed in the old mice receiving transplants from young donors, demonstrating a crucial role of donor age in FMT efficacy. The differences in significance of experimental results across both Old and Young groups is summarized in Table 1.

Gut microbial population is known to change with age [61]. Aging is associated with decreased microbial diversity that also correlates with the number of concomitant diseases, and number of medications used [62]. As we have shown, ‘old’ 5xFAD mice treated with microbial transplants from young WT mice (Old Tg-FY) display a trend of normalizing behaviors towards or superseding wildtype measurements. Whilst transplants from age-matched WT mice also afforded beneficial effects in old Tg mice (Old Tg-FO), overall cognitive measures (Figure 5a) were clearly superior in the Old Tg-FY mice, underscoring the importance of donor age, suggesting improved FMT outcomes with younger donors. Future investigations in both animal models and humans should focus on the effects afforded by transplanting microbial material from younger compared to older donors and should also investigate inflammatory changes that occur as a result of decreasing pathology in the AD brain.

This study has several limitations. A general limitation would be the murine model, which is not directly generalizable to human disease models. However, other studies have shown the use of FMT in humans [28,29,30,31] and our study adds value by providing a proof-of-concept of donor age-dependent effects of intervention on Alzheimer pathology. A more specific limitation would be the period of treatment (7 days) and incubation (21 days). A longer period of treatment or incubation following FMT may have elicited more desirable results. Future studies could vary incubation periods and analyze intestinal microbial and metabolic composition to further elucidate pathways that allow FMT to reduce AD pathology.

## 5. Conclusions

We report significant changes in amyloid plaque burden and cognitive measures in the 5xFAD mouse model of Alzheimer’s disease following a 7-day fecal microbiota transplantation. A complete reversal in cognitive measures in 32-week-old (at commencement of treatment) 5xFAD mice who received FMT from 8–10-week-old (young) wildtype donors and normalization in cognitive measures accompanied by decreases in amyloid plaque load in 32-week-old transgenic mice receiving FMT from age-matched donors suggest a novel treatment strategy for Alzheimer’s disease that is easily translatable to human patients and supports other studies that show that clearing amyloid plaques in the brain is associated with cognitive improvements. We also demonstrate that donor age plays an important role in fecal microbiota transplantation that must be further investigated.

## Figures and Tables

**Table 1:** Summary of difference in significance across experimental groups for behavioral studies experimental measures and Aβ loading. Young Tg-FY: Young Transgenic-Fed Young, Young Tg-Control: Young Transgenic Control, Old Tg-FY: Old Transgenic-Fed Young, Old Tg-FO: Old Transgenic-Fed Old, EPM: Elevated Plus Maze, NOR: Novel Object Recognition, FAY: Forced Alternation Y-maze.

Experimental Measure	Old Tg-FO	Old Tg-FY	Young Tg-FY
EPM	*p* = 0.687	*p* = 0.056	*p* = 0.470
NOR	*p* = 0.065	*p* = 0.002	*p* = 0.409
FAY	*p* = 0.046	*p* = 0.012	*p* = 0.505
Aβ (amyloid load; plaque number)	*p* = 0.010; *p* = 0.060	*p* = 0.010; *p* = 0.045	*p* = 0.359

## Data Availability

Analyzed data is available in the main text. All data is available as on the Research Data Store (RDS) of the University of Sydney. Further information and requests for resources should be directed to the Lead Contact, Damian Holsinger (damian.holsinger@sydney.edu.au).
